# Bonobos and chimpanzees remember familiar conspecifics for decades

**DOI:** 10.1073/pnas.2304903120

**Published:** 2023-12-18

**Authors:** Laura S. Lewis, Erin G. Wessling, Fumihiro Kano, Jeroen M. G. Stevens, Josep Call, Christopher Krupenye

**Affiliations:** ^a^Department of Human Evolutionary Biology, Harvard University, Cambridge, MA 02138; ^b^School of Psychology & Neuroscience, University of St Andrews, St Andrews KY16 9AX, United Kingdom; ^c^Kumamoto Sanctuary, Wildlife Research Center, Kyoto University, Kumamoto 862-0911, Japan; ^d^Center for the Advanced Study of Collective Behavior, University of Konstanz, Konstanz 78457, Germany; ^e^Behavioural Ecology and Ecophysiology, Department of Biology, University of Antwerp, Antwerp BE-2000, Belgium; ^f^Centre for Research and Conservation, Royal Zoological Society of Antwerp, Antwerp 2018, Belgium; ^g^Department of Psychological & Brain Sciences, Johns Hopkins University, Baltimore, MD 21218

**Keywords:** long-term social memory, social relationships, eye-tracking, primates, cognitive evolution

## Abstract

Recognition and memory of familiar conspecifics provides the foundation for complex sociality and is vital to navigating an unpredictable social world [Tibbetts and Dale, *Trends Ecol. Evol.* **22**, 529–537 (2007)]. Human social memory incorporates content about interactions and relationships and can last for decades [Sherry and Schacter, *Psychol. Rev.* **94**, 439–454 (1987)]. Long-term social memory likely played a key role throughout human evolution, as our ancestors increasingly built relationships that operated across distant space and time [Malone *et al*., *Int. J. Primatol.* **33**, 1251–1277 (2012)]. Although individual recognition is widespread among animals and sometimes lasts for years, little is known about social memory in nonhuman apes and the shared evolutionary foundations of human social memory. In a preferential-looking eye-tracking task, we presented chimpanzees and bonobos (*N* = 26) with side-by-side images of a previous groupmate and a conspecific stranger of the same sex. Apes’ attention was biased toward former groupmates, indicating long-term memory for past social partners. The strength of biases toward former groupmates was not impacted by the duration apart, and our results suggest that recognition may persist for at least 26 y beyond separation. We also found significant but weak evidence that, like humans, apes may remember the quality or content of these past relationships: apes’ looking biases were stronger for individuals with whom they had more positive histories of social interaction. Long-lasting social memory likely provided key foundations for the evolution of human culture and sociality as they extended across time, space, and group boundaries.

## Significance

While human social memory lasts decades and tracks relationships, less is known about nonhuman ape long-term memory.We present evidence that both chimpanzees and bonobos recognize the faces of familiar conspecifics even after many years of separation. An eye-tracking task revealed that apes’ attention was biased toward former groupmates over strangers, and this pattern may persist for at least 26 y beyond separation. Apes’ memory may also represent the quality of their social relationships: Apes looked longer toward individuals with whom they had more positive relationships.Thus, critical properties of human memory may reflect deep homologies with other apes, likely providing the foundation for the emergence of complex cooperative relationships that operate across long time-scales.

Complex sociality requires individuals to recognize and remember conspecifics across space and time (1–13). Long-term memory for social partners and interactions allows animals to build individual relationships, strategically navigate dominance hierarchies and alliances, and avoid hostile interactions (1, 2, 14). Humans are notable within the animal kingdom for our ability to remember others’ names and faces and track information about social roles, groups, and relationships for decades (4–9, 15–17). Yet, the phylogenetic origins of our rich social memory remain unclear. Apes have shown long-term memory for past physical events in experimental contexts, and some monkey and ape species track third-party relationships and the spatiotemporal patterns of fruiting trees across time (18–22). Our closest living relatives, chimpanzees (*Pan troglodytes*) and bonobos (*Pan paniscus*), also exhibit complex intergroup dynamics, build friendships and alliances, and show evidence of third-party knowledge, despite sometimes going days without seeing members of their communities (18, 23–33). However, while there is evidence that some primates remember familiar conspecifics for multiple years, this memory may be limited in our very closest relatives, as bonobos cease to display vocal recognition of former groupmates beyond 5 y of separation (34–36). To our knowledge, there are currently no studies on multiyear social memory in chimpanzees. Moreover, evidence of the most enduring nonhuman social memory [20 y in dolphins: (13)] or of memory that encodes information about relationship quality [ravens: (37)] comes only from distant relatives of humans, possibly indicating convergent evolution. Additional data from nonhuman apes are therefore essential to determine which features of social memory are derived human traits and which are phylogenetically basal homologies with other primates.

**Fig. 1.**
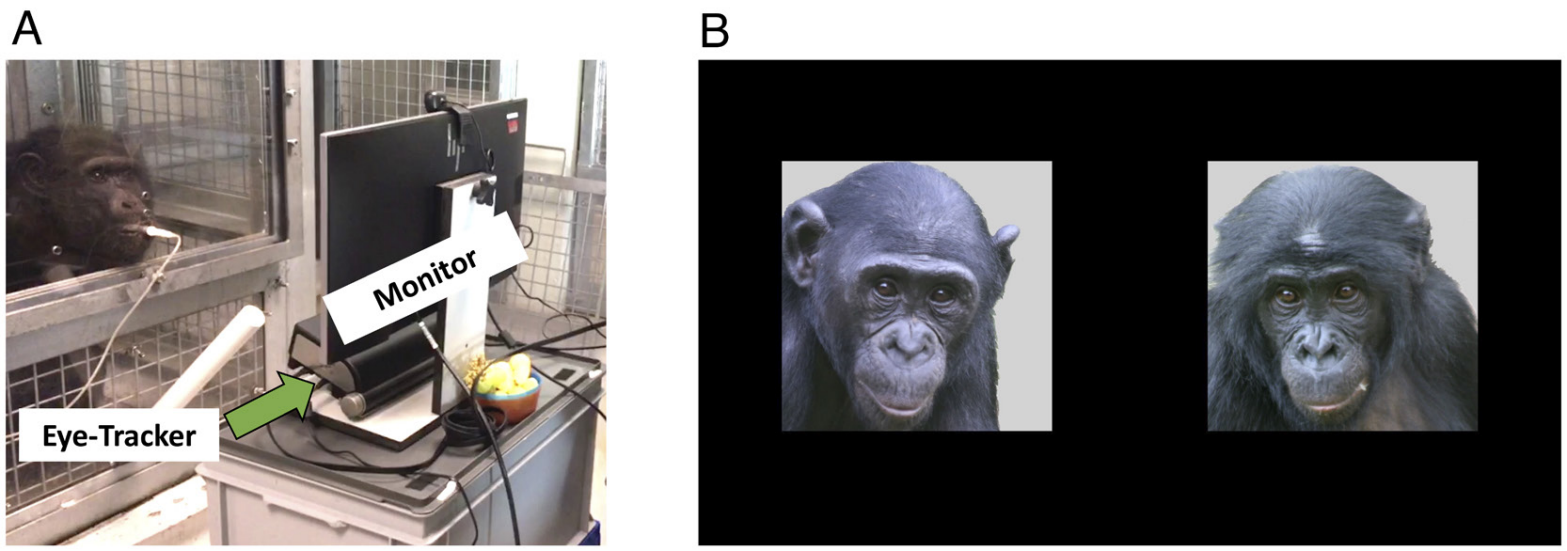
Experiment details. (*A*) Experimental setup at Edinburgh Zoo, showing a chimpanzee volunteer drinking juice while attending to a computer monitor where his gaze is noninvasively recorded with a remote eye-tracker. (*B*) Example of side-by-side images of a former groupmate and an unfamiliar conspecific of the same sex for bonobo trials at the Planckendael Zoo population.

To clarify the shared evolutionary foundations of human social memory, we tested both bonobos and chimpanzees, across four populations in three countries, on a preferential-looking eye-tracking task examining memory for conspecific faces (*N* = 26, 15 females, aged 4–46 y, average age = 25.9; see *SI Appendix*, Tables S1 and S2 for further details). While their gaze was noninvasively recorded, zoo- and sanctuary-housed apes viewed side-by-side same-sex images of a conspecific stranger and a former groupmate who had either died or been transferred to another facility (Fig. 1). The time since subjects had last seen previous groupmates was at least 9 mo but varied considerably across dyads (*SI Appendix*, Table S3). We first tested the hypothesis that apes possess long-term memory for conspecific faces, which predicted a consistent looking bias toward former groupmates over strangers. We then investigated several key properties of ape social memory.

## Results

### Apes Exhibit Years-long Memory for the Faces of Former Groupmates.

To examine individual recognition, we followed past preferential looking work (e.g., refs. 38–40) and constructed two metrics of attentional bias toward former groupmates. For each 3-s trial, we identified fixations (i.e., the maintenance of gaze on a location) to each avatar. We then calculated a raw difference score (i.e., [sum of fixations to former groupmate] minus [sum of fixations to unfamiliar stranger]) and a proportional Differential Looking Score (DLS; i.e., [fixations to groupmate minus fixations to stranger] divided by [fixations to groupmate plus fixations to stranger]) (41). We used both raw difference scores and DLS because they each capture biases in looking patterns with slightly different focuses: Raw difference scores reveal absolute differences in attention, while the DLS amplifies biases even on trials with brief attention times. Given high consistency in direction and effect sizes of analyses involving each metric, for conciseness, we only report raw difference score analyses in the main text and DLS analyses in *SI Appendix*.

In Model Set 1, we investigated attentional biases toward kin and nonkin groupmates (kin defined as having a relatedness coefficient *r* ≥ 0.25, derived from studbook data). We used linear mixed effects models, fitted separately with raw difference score and DLS as dependent variables. Critically, as these metrics were centered at zero, a model intercept that was significantly different from zero indicated a significant bias in attention toward previous groupmates or unfamiliar strangers for trials within the reference category (a positive intercept term indicated that attention to the former groupmate was greater than attention to the stranger; a negative intercept term indicated the opposite). We included a single test predictor (indicating whether the former groupmate was the subject’s kin vs. nonkin; all strangers were nonkin), a single control predictor (z-transformed trial number, to control for potential habituation across trials), random intercepts of subject identity, and the IDs of the familiar and unfamiliar avatars (as a single dyad ID). The null model included the single control predictor (trial number) and the random intercepts (subject ID, avatar dyad ID).

**Fig. 2.**
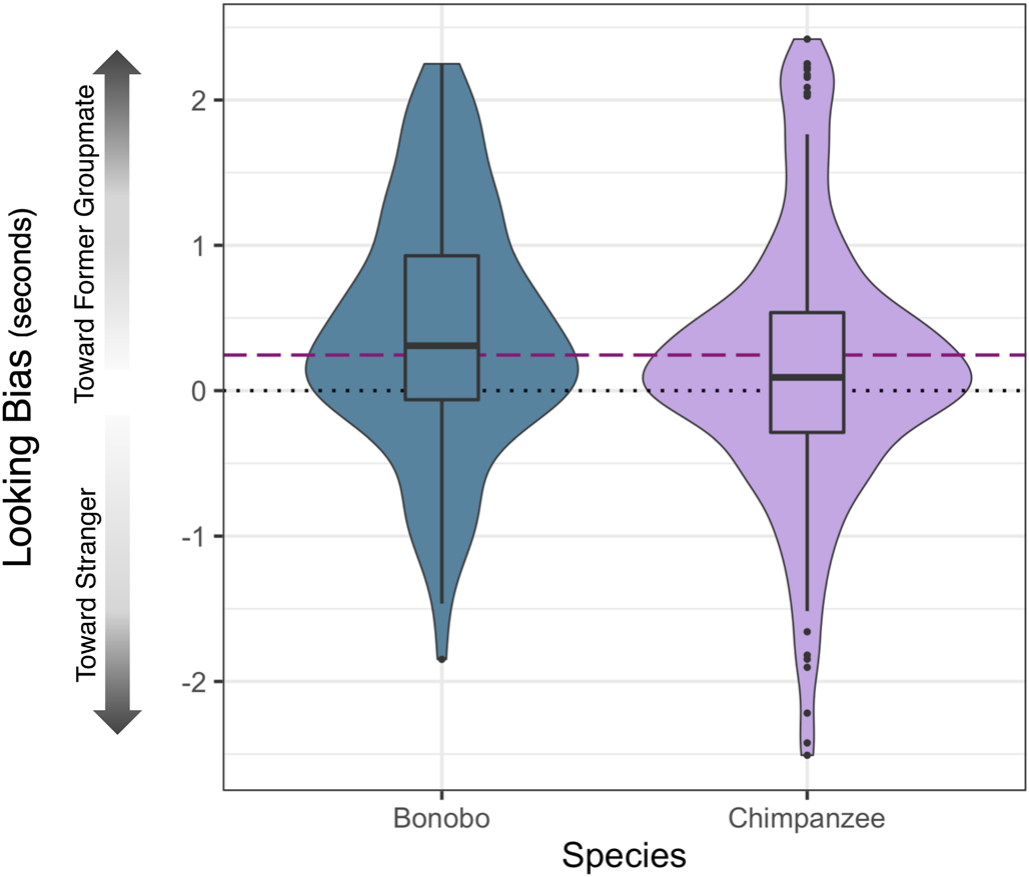
Biases in raw difference scores toward previous groupmates relative to strangers, by population. These data include kin and nonkin data and only include trials from the refined dataset (with looks to both avatars). Kumamoto apes exhibit significantly stronger looking biases than European apes (Model Set 2). The purple dashed line denotes raw difference scores Model 1 intercept (with restricted dataset) with kin as the reference category; the black dotted line denotes chance (equal looking to previous groupmate and unfamiliar conspecific). Boxes denote the interquartile range (IQR, from 25th percentile to 75th percentile), and middle lines denote population means.

The full-null model comparison for Model 1a was not significant (χ^2^ = 0.0001, *P* = 0.992), indicating that our single test predictor—whether the former groupmate was the subject’s kin—did not influence looking biases. Kin recognition mechanisms distinct from memory are therefore unlikely to explain biases in social attention. Model 1a revealed a positive and significant intercept term with nonkin as the reference category of the kin vs. nonkin test effect (estimate = 0.244 ± 0.072 (SE), *P* = 0.002), indicating a significant looking bias toward nonkin former groupmates over strangers (*SI Appendix*, Tables S4 and S5). Specifically, apes on average looked for 0.24 s (~11%) longer at images of former groupmates relative to strangers. Within the same Model 1a, when kin was set as the reference category, the intercept term was marginally significant (estimate = 0.245 ± 0.137 (SE), *P* = 0.076). These findings demonstrate that bonobos and chimpanzees recognize former groupmates, even after years of separation. In the most extreme case, bonobo Louise had not seen her sister Loretta nor nephew Erin for over 26 y at the time of testing. Strikingly, she showed a robust attentional bias toward both Loretta and Erin (across eight trials, average raw difference score for Loretta: 0.775; for Erin: 0.407). The biases on these trials were significantly greater than chance (*P* = 0.012, one sample *t*-test) and exceeded model averages. Interestingly, our results seem to be partially driven by our more research-experienced ape populations, with only the chimpanzees and bonobos at Kumamoto Sanctuary (including Louise) showing robust biases toward former groupmates (*SI Appendix* and Fig. 2).

In our second set of analyses (Model Set 2), we investigated memory degradation and whether dominance patterns or social relationships shaped social memory by examining predictors of attentional biases toward nonkin (to exclude confounds of kinship). However, we found the distribution of model residuals in Model 2a to violate assumptions of normality. Upon visual inspection, we identified residual skews that derived from a large number of trials (196 of 463 nonkin trials) in which apes only fixated on a single avatar rather than both. Given this issue, and the fact that trials in which subjects have not observed both avatars may be less informative with regard to discrimination *between* avatars, we subsequently removed these trials where both avatars had not been viewed (see refs. 42 and 43 for similar exclusion criteria and *Methods* for details). The remaining 267 nonkin trials in which subjects looked at both avatars constituted our *refined dataset*, with which we ran Model 2b. We also reran Model 1a with a refined dataset that applied the same exclusion criteria (leaving 314 of 524 trials with kin and nonkin) and found no difference to model inference (Model 1b, see *SI Appendix*, Tables S6 and S7). As the majority of removed trials (111 of 210 trials) consisted of fixation only upon the familiar groupmate, we note that their removal makes Model 1b even more conservative against our predictions.

**Fig. 3.**
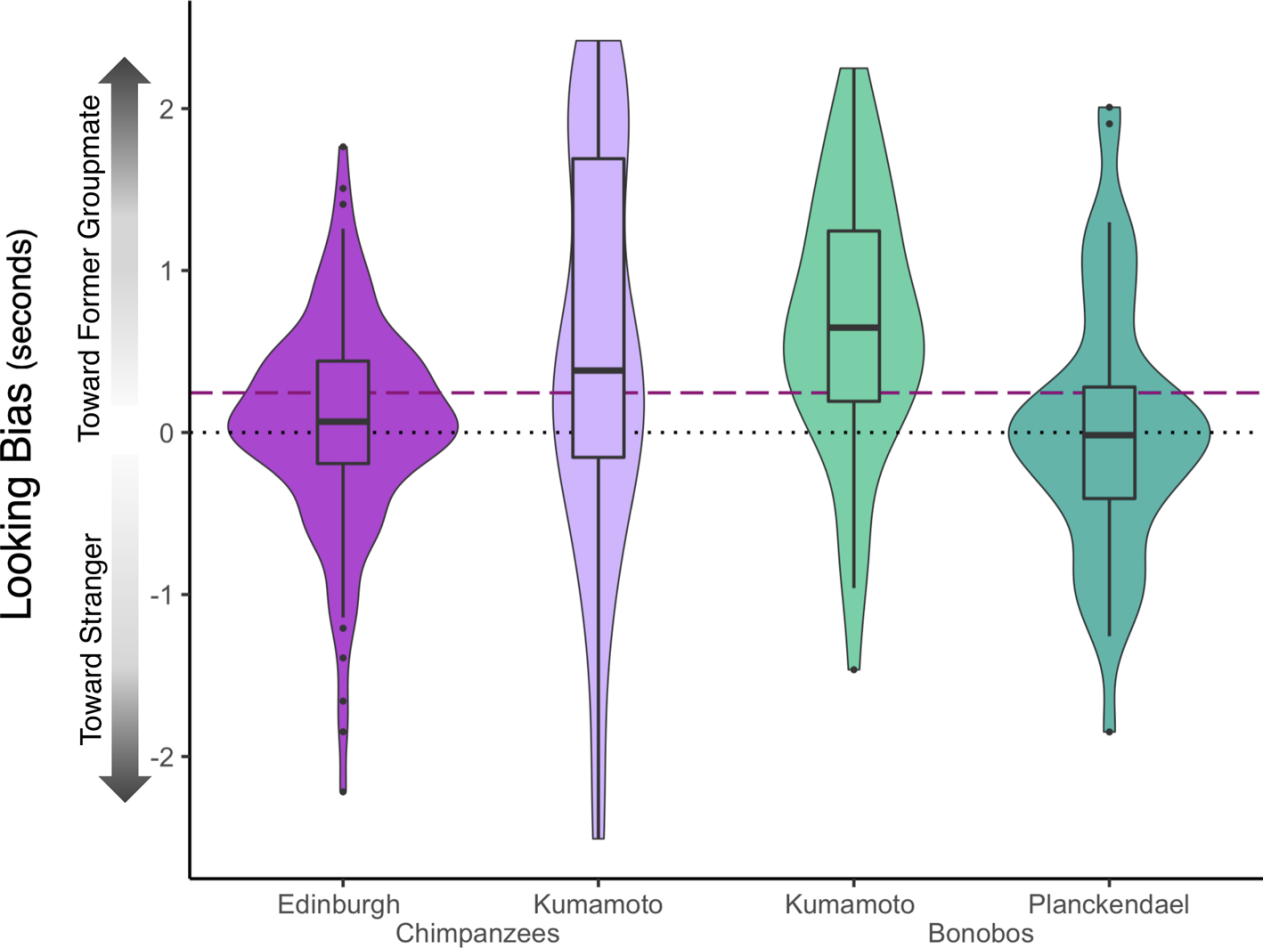
Biases in attention toward former groupmates relative to strangers. Distribution of raw difference scores for each species, calculated as the sum of fixations (in seconds) to the former groupmate minus the sum of fixations to the unfamiliar stranger. These data include kin and nonkin data and only include trials from the refined dataset (with looks to both avatars). We find a looking bias toward former groupmates (Model Set 1) and no difference between species (Model Set 2). The purple dashed line denotes raw difference scores Model 1 intercept (with restricted dataset) with kin as the reference category; the black dotted line denotes chance (equal attention to previous groupmate and unfamiliar conspecific). Boxes denote the interquartile range (IQR, from 25th percentile to 75th percentile), and middle lines denote species means.

Refitting Model 1b to the refined dataset produced identical results, as we found no significant difference in looking patterns between trials with former groupmates that were kin vs. nonkin (estimate = 0.0003 ± 0.153 (SE), *P* = 0.998). The intercept term also remained significant for nonkin trials (estimate = 0.248 ± 0.084 (SE), *P* = 0.006, see Fig. 3), with apes fixating on average for 0.25 s (~14%) longer toward images of former groupmates than toward strangers. For kin trials, we again found a positive trend for the intercept term (estimate = 0.248 ± 0.154 (SE), *P* = 0.109). This looking bias toward kin was significant in the Kumamoto Sanctuary apes (*SI Appendix*). Thus, across both full and reduced datasets, we find consistent evidence that bonobos and chimpanzees recognize the faces of conspecifics that they have not seen in years.

### Predictors of Attentional Biases.

Next, we examined predictors of attentional bias using the refined dataset. We focused specifically on nonkin trials for three reasons: to further exclude kin recognition as an explanation for bias; because the small number of kin trials limited power for examining sources of variation; and because nonkin relations may meaningfully differ from kin relations (44–46). We fitted Model Set 2 separately with raw difference score and DLS as dependent measures and included the same random intercepts as Model 1a. In Model Set 2, we tested two hypotheses about ape social memory. First, to examine memory degradation over time, we included as a test predictor the time interval between the start of this experiment and the subject’s last co-housing with the former groupmate (“time apart”). Second, we tested the hypothesis that the nature of social relationships shapes memory in apes, as it does in humans. Accordingly, we included as predictors ratings of the frequency of positive interactions between the subject and former groupmate, ratings of the frequency of negative interactions, and relative dominance (all at the time of separation). Because historical observational data were not available, these three relationship metrics were rated by caretakers and researchers familiar with the subjects and former groupmates. Raters were blind to eye-tracking data and had high interrater reliability (see *SI Appendix*, Table S8 for interrater reliability scores). We also included an interaction between species and sex of the conspecific avatars, in line with the recent finding that apes preferentially attend to familiar members of the more dominant sex [i.e., male chimpanzees, female bonobos: (38)]. Finally, we included several control predictors to account for their potential impact upon looking biases: duration of co-housing (to control for general familiarity between the subject and former groupmate), subject sex, subject age at the time of separation, age class of the former groupmate (to control for biases toward younger or older conspecifics), and trial number (z-transformed). We also included population, dummy-coded as “European apes” (Edinburgh chimpanzees, Planckendael bonobos) and “Kumamoto apes” (Kumamoto chimpanzees and bonobos), as previous research identified differences between conspecifics of these populations (38). We compared these models to null models excluding only the aforementioned test predictors (duration apart and the three predictors of relationship) using a likelihood ratio test.

**Fig. 4.**
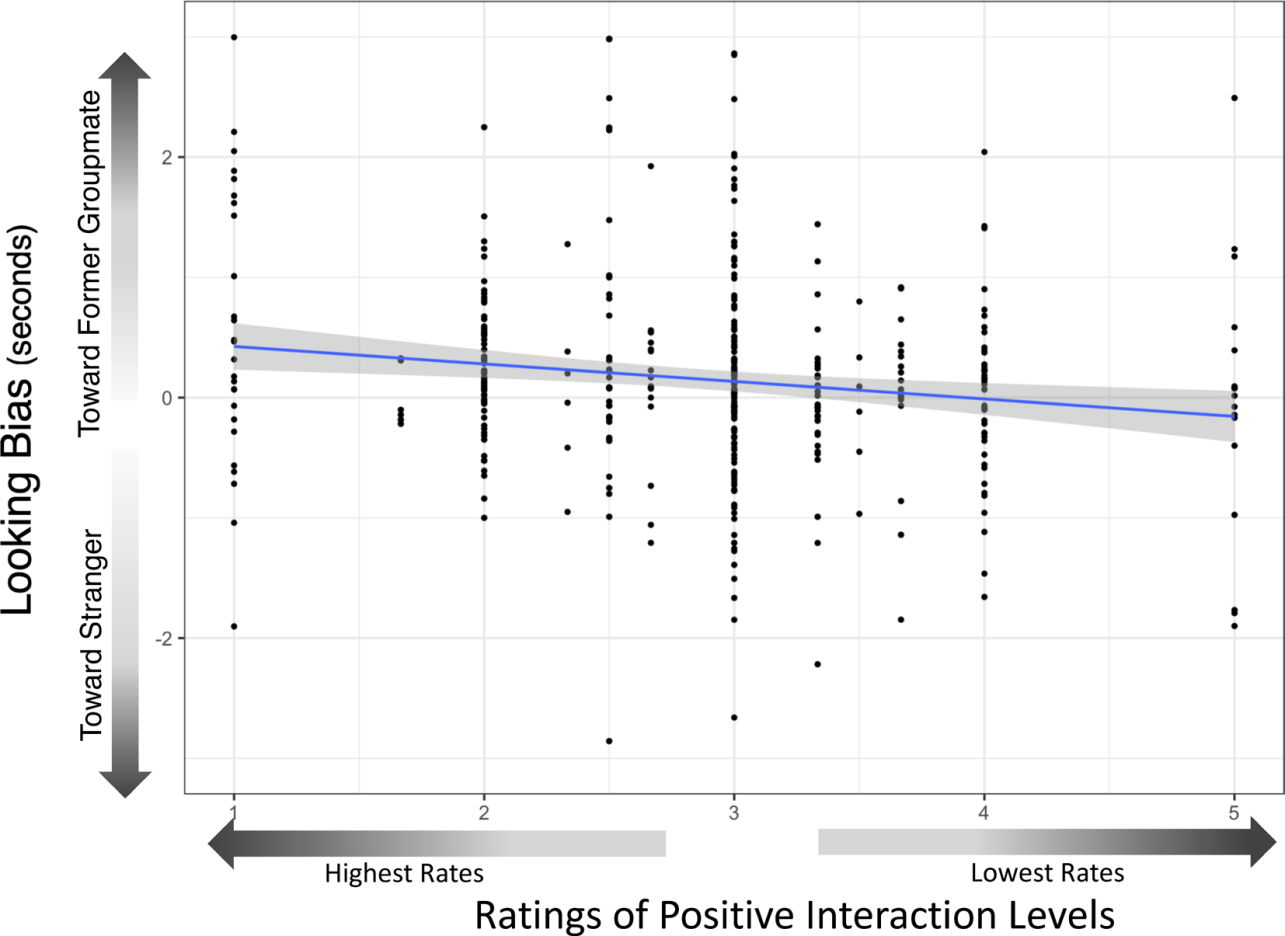
Effect of former relationship rating on looking bias. This figure depicts the relationship between looking bias (raw difference score) and rates of positive social interaction at the time when subjects had last seen former groupmates, in the restricted dataset. All data represent nonkin relationships. Positive interaction metrics were rated by animal caretakers, who were blind to looking data, and exhibited high interrater reliability. Apes showed significantly stronger looking biases toward former groupmates with whom they previously had higher rates of positive interaction. The blue line represents the model regression line, and the gray box denotes the 95% confidence region.

Full-null model comparisons for Model 2b were not significant (χ^2^ = 5.045, *P* = 0.538). However, we explored the contribution of the individual test predictors as their concurrent exclusion from the null model in the full-null model comparison masks their individual effects (47). There was no significant interaction between species and avatar sex, and, therefore, we dropped this interaction term and refitted the models. Critically, the effect of time apart was not significant, suggesting that memory did not significantly differ across the durations captured within the nonkin dataset (maximum = 9.54 y). However, Model 2b with raw difference scores revealed that apes showed significantly stronger attentional biases toward groupmates with whom they previously had higher frequencies of positive interaction (estimate = −0.159 ± 0.081 (SE), *P* = 0.049; see Fig. 4), suggesting that some component of positive social relationships is represented in apes’ long-term social memory. We also found an effect of trial number (estimate = −0.163 ± 0.054 (SE), *P* = 0.003), indicating a decline in overall attention toward avatars as trials progressed (*SI Appendix*, Tables S9–S11). Finally, we found an effect of population on looking bias (estimate = 0.47 ± 0.167 (SE), *P* = 0.006), indicating Kumamoto apes’ greater attention toward former groupmates. In a final set of exploratory analyses, we restricted the sample to trials in which subjects attended to the screen above the duration required for human face recognition [>400 ms (48)], and reran Model Sets 1 and 2. All effects increased and remained significant in these trials in which subjects were presumably more engaged with the task (*SI Appendix*, Tables S18–S21). Most notably, the pattern of stronger attentional biases toward more positive social partners (i.e., those with whom subjects previously had higher frequencies of positive interaction) was much clearer (estimate = −0.239 ± 0.089 (SE), *P* = 0.008; *SI Appendix*, Table S20).

## Discussion

Our results support the hypothesis that bonobos and chimpanzees possess an enduring memory for previous social partners. Apes demonstrated biases in attention toward the faces of former groupmates as compared to the faces of strangers, with some of the strongest biases observed among individuals who had not seen each other for decades. We found no evidence that recognition memory substantially declines for the time windows captured within our analyses, as duration apart did not impact attentional biases. The results of Model 2b indicate that apes likely have memory for nonkin lasting at least 9.5 y, and the full dataset from Model 1b suggests that apes may remember social partners for much longer, up to 26 y—a large proportion of their 40- to 60-y lifespans (49). Although more data are needed to determine whether great ape memory lasts beyond 26 y, these results indicate that, for at least some nonhuman great apes, the longevity of social memory may be relatively similar to that of humans, which begins to decline after ~15 y but can persist 48 y beyond separation (5). Nonhuman social memory lasting more than a few years has only been documented in dolphins, which recognize others’ vocalizations for 20 y. Our results therefore provide evidence for the longest-lasting nonhuman social memory documented to date. Critically, our various lines of evidence from both chimpanzees *and* bonobos greatly exceed the 5.5-y duration of vocal recognition previously identified in bonobos (36). Our results thus help to pinpoint the likely phylogenetic timing of the emergence of long-term social memory in humans.

Apes’ attentional biases not only appear to reflect basic features of familiarity but may also track complex properties of their social relationships, although this finding should be interpreted cautiously and future work should aim to further clarify these properties. We found a weak but significant pattern of stronger looking bias toward individuals with whom the apes previously had more positive relationships (that became much stronger when examining only trials with higher screen engagement: *SI Appendix*, Tables S20 and S21). This finding is important for our understanding of how social relationships may shape memory in primates, as similar evidence has only been documented in one other species: ravens (37). Whereas ravens distinguished classes of familiar conspecifics [former affiliates vs. nonaffiliates, (26)], in our study, a continuous metric of relationship quality predicted apes’ looking biases. Rather than reflecting class-level categorization, our results may indicate that apes possess a more nuanced relationship representation. We advocate for the replication of this effect in future research, given its significance to major debates about primate social cognition. For example, as positive relationships, including friendships, are an important universal feature of the social lives of *Hominini* (45, 50, 51), our finding about the representation of social relationships may also help address historical questions about the cognitive mechanisms that support reciprocity in primates (33): while we cannot know whether primates are carefully scorekeeping by tracking individual past experiences, as is involved in calculated reciprocity, our data suggest that they may at least represent some aggregate positive-ness of each of their individual relationships based on their history of interaction.

We also found stronger attentional biases toward former groupmates in the apes living at Kumamoto Sanctuary relative to those in European zoos. One possible explanation is that juvenile apes, only present in the European samples (*SI Appendix*, Table S1), may have poorer memory capacities (13); however, the removal of these three individuals from analyses did not impact populational differences. It could also be that individuals with smaller conspecific social networks (past and present) show clearer memory because they have fewer individuals to remember. Indeed, at the time of testing, both Kumamoto groups were smaller than both European groups (*SI Appendix*, Table S2); however, this may not account for historical cumulative group membership experienced by these apes. Therefore, to examine the apes’ historic social networks, we compiled a list of all groupmates that each participant had ever lived with, past and present (*SI Appendix*, Table S12), and evaluated this list to determine whether there were large differences in total lifetime numbers of groupmates. Although the Edinburgh chimpanzees have lived with larger numbers of conspecifics than have the Kumamoto chimpanzees (excluding nongroupmate chimpanzees at Kumamoto Sanctuary), the Planckendael and Kumamoto bonobos have total lifetime social networks of highly similar sizes. Overall, then, past and present social network size does not seem to fully explain the stronger attentional biases toward former groupmates seen in the Kumamoto apes relative to the European apes.

Instead, it may be that the difference stems from differences in exposure to screen-based eye-tracking experiments and perhaps to research context. The Kumamoto apes are reliably separated for testing, reducing distractions, whereas separations do not occur at Edinburgh Zoo and occur to a lesser extent at Planckendael. Moreover, the Kumamoto apes have participated in many eye-tracking (and touchscreen) experiments, whereas this technology is newer for the European populations. It may be that the Kumamoto Sanctuary apes have learned to attend more robustly to screens and are also more familiar with viewing pictures and discriminating between individuals in pictures compared to the apes living in European zoos. In support of this view, the Kumamoto apes looked substantially longer (1.895 s ± 0.677) at stimuli (total fixations to both avatars) than did the European apes (1.037 s ± 0.656), suggesting that they indeed engaged more fully with the task (*SI Appendix*, Table S11).

While the majority of past ape cognition research presents data from a single site per species, the inclusion of multiple sites was key to identifying these population differences. Where possible, we encourage future eye-tracking research to also include multiple conspecific ape populations to help clarify the extent (and drivers) of population differences in looking biases. Furthermore, testing a greater number of individuals within each population (including a broader range of ages and developmental stages) and an even larger number of populations would allow for a stronger survey of variation in long-term social memory between individuals and populations. Training with eye-tracking and computerized setups, as in the Kumamoto sample (and potentially exclusion of low-attention trials), may also help to improve attention and reduce the inherent noisiness of gaze data.

High interrater reliability lends confidence to our relationship metrics, but future work can extend these findings with direct observational data. We carefully controlled for the identities and gaze direction of the avatars and counterbalanced their positions (right vs. left side of the screen) across trials. We used the maximum number of previous groupmates for which suitable photos were available (good quality, and originating shortly before the dyad’s separation), demonstrating that these effects generalize across apes and groupmates. We analyzed both raw difference scores to reveal absolute differences in attention and DLS which amplifies biases even on trials with brief attention times. To control for biases toward specific individuals, the images of previous groupmates for the Edinburgh chimpanzees and the Planckendael bonobos were used as unfamiliar images for the Kumamoto chimpanzees and bonobos, respectively. Finally, we controlled for kin recognition mechanisms in Model Set 2 by excluding any previous groupmates that had a relatedness coefficient *r* ≥ 0.25 with a subject.

Although our results suggest that nonhuman great apes’ long-term memory may be shaped by social relationships, we cannot say whether the underlying mechanisms are specific to social information or are common to those involved in memory for objects or events. It is clear, however, that attention and memory are influenced by familiarity and social factors (38, 52, 53). Our results also do not speak to whether information beyond facial identity and relationship quality is encoded in these representations, such as auditory or third-party information. However, previous work (54–57) suggests that primates’ representations of others’ identities are not specific to a single modality; rather, these representations integrate both visual and auditory information. The results from this study extend recent findings that bonobos recognize the vocalizations of previous groupmates for at least 5 y (36). Our work also clarifies that apes’ social memory integrates visual information. The greater longevity of memory for faces raises the intriguing possibility that visual components of recognition memory may be favored in memory, or more robust to degradation than auditory components. Alternatively, perceptual features of identity may be more stable in adult apes’ faces than in their vocalizations, although this remains to be tested. Notably, vocal signatures of identity remain stable in at least some species, as dolphins recognize others’ vocalizations for at least two decades (13). These insights are possible only through integration of data on visual and vocal recognition.

Evidence for multiyear social memory has been demonstrated in just a handful of species, many of which exhibit complex and/or fission-fusion social organization [e.g., fur seals: (11); sheep: (10); bottlenose dolphins: (13); elephants: (12); Japanese macaques: (34); orangutans: (35); bonobos: (36)]. The phylogenetic distance between these species raises the question of whether their similar capacities for multiyear social memory owe to convergent evolution or to deep phylogenetic homology. A paucity of data on the longevity of social memory and its sensitivity to relationship information in these species limits inference about the underlying cognitive mechanisms or the socioecological drivers of variation in memory capacities. Further work on more closely related species, especially with differing degrees of sociality, could help resolve these gaps (e.g., ref. 14).

Our findings in humans’ closest relatives support the hypothesis that human mechanisms for decades-long social memory were likely present at least 6–9 mya in our last common ancestor. These ancient capacities likely provided key foundations for the emergence of uniquely human forms of interaction and cooperation, such as intergroup trade appearing at least 500 KYA, as our species expanded into distant environments and experienced extended periods away from familiar individuals (57–59). Long-lasting memory for groupmates, especially close social partners, thus may have aided in the stability of early humans’ dyadic relationships and facilitated the evolution of cooperative cultural systems that extend across time, space, and group boundaries.

## Methods

### Subjects.

We tested twenty-six ape participants from four groups across three locations: Edinburgh Zoo, Scotland (9 chimpanzees: 3 females, 6 males), Kumamoto Sanctuary, Japan (6 chimpanzees: 5 females, 1 male; 6 bonobos: 4 females, 2 males), and Planckendael Zoo, Belgium (6 bonobos: 3 females, 3 males). Subjects ranged in age from 2 to 46 y (bonobo mean = 21.9 ± 13.8 y (SD); chimpanzee mean = 27.1 ± 10.6 y (SD).

### Ethical Note.

Experimental protocols adhered to the School of Psychology and Neuroscience Animal Ethics Committee at the University of St Andrews and to approval by each participating animal care institution. Edinburgh and Kumamoto Sanctuary participants were tested in the testing rooms prepared for each species, whereas the Planckendael participants were tested in their large indoor enclosure. Apes’ daily participation in this study was completely voluntary. They received regular feedings and daily enrichment and had ad libitum access to water. Animal husbandry and research protocol complied with international standards (the Weatherall report, The use of nonhuman primates in research) and institutional guidelines [Kumamoto Sanctuary: Wildlife Research Center Guide for the animal research ethics; Edinburgh and Planckendael Zoos: EAZA Minimum standards for the accommodation and care of animals in zoos and aquaria; WAZA Ethical guidelines for the conduct of research on animals by zoos and aquariums; Guidelines for the treatment of animals in behavioral research and teaching (ASAB/ABS)].

### Apparatus.

We applied established eye-tracking procedures and comparable setups across facilities (38, 60–62). Images were presented to apes through a transparent polycarbonate or acrylic panel on a 23″ LCD monitor just outside of their enclosures at a distance of approximately 60 cm between the display and the subject’s face. Subjects’ eye movements were noninvasively recorded via an infrared eye-tracker (X120 in Edinburgh and Planckendael, X300 in Kumamoto, Tobii Technology AB), positioned directly below the monitor, which mapped their gaze onto the stimulus images. Stimulus presentation and data collection were controlled using Tobii Studio. Apes were provided a small amount of diluted fruit juice (provided irrespective of viewing patterns) delivered through a plastic nozzle positioned on the transparent panel, directly in front of the eye-tracker. Juice encourages apes to voluntarily position themselves at the eye-tracking setup and allows us to minimize head movements and optimize corneal reflection measurements (Fig. 1).

Before testing, we conducted a two-point automated calibration for each ape subject by presenting a small video clip (and often a small piece of fruit held up in front of the screen) on each reference point. This two-point calibration procedure is frequently used in eye-tracking studies with great apes to provide high-quality data and minimize the potential loss of subjects who would not reliably attend to a greater number of calibration points (60, 63, 64). After we obtained each calibration, we manually checked the accuracy of the calibration using nine points on the screen and repeated the calibration process if the calibration was deemed inaccurate based on visual inspection. Each subject’s unique calibration was used throughout the entire testing period. Prior to the start of every session, the calibration accuracy was checked with at least one of the nine points and calibration was repeated when necessary. Calibration errors are typically less than a degree with this procedure, and any error of this size will not impact the ability to determine preferential looking to images (61).

### Stimuli.

The stimuli in this study consisted of static 600 × 600 pixel close-up color photographs of forward-facing conspecific faces with neutral facial expressions (hereafter referred to as “avatars”) and a gray background (Fig. 1). Each trial featured two images, one of a previous groupmate and another of an unfamiliar conspecific, on the center-left and center-right regions of a black 1920 × 1080 pixel screen (locations counterbalanced across trials). The distance between the right-most edge of the left avatar and the left-most edge of the right avatar was 347 pixels. Previous groupmates included conspecific individuals who had previously lived with the subjects’ group but had either died or been transferred to a new group one or more years prior to testing. We used photos from as close as possible to the time when subjects last saw previous groupmates. “Unfamiliar” conspecifics included only individuals who had never been housed at the same institution as the subject, according to studbook and institutional data. Within trials, the images were sex-matched and age class-matched, and the face and eye direction, brightness, contrast, and blurriness of photographs were kept as consistent as possible across stimuli. The images of Loi, Tsubaki, Pearl, Misaki, and Mizuki were compressed to 30% of their original image to match the other images in the Kumamoto chimpanzee and bonobo sets. In some cases, photos of previous groupmates at one facility were used as unfamiliar conspecifics at the other, and vice versa. This allowed us to further control for any perceptual differences across stimuli that could bias attention.

Chimpanzees at the Edinburgh Zoo and Kumamoto Sanctuary experienced two stimuli sets, one of adult female avatars and the other of adult male avatars. For the Edinburgh Zoo chimpanzees, each stimulus set consisted of three images of previous adult groupmates and three images of unfamiliar adult conspecifics. The Kumamoto Sanctuary chimpanzees only had one previous female groupmate and one previous male groupmate. Therefore, to maintain even counterbalancing of image presentation and equal degrees of novelty across stimuli (i.e., the images of true previous groupmates did not appear more frequently than the unfamiliar conspecific images with which they were paired), we only presented images of true previous groupmates (one image per previous groupmate), and then filled the remaining “previous groupmate” trials with pairs of images of unfamiliar individuals (matched for age/sex classes of other images in the trial). Trials that contained two images of unfamiliar conspecifics, which we call “fake trials,” were excluded from all subsequent analyses (*SI Appendix*, Table S13).

The Kumamoto Sanctuary bonobos were presented with two sets of female stimuli and two sets of male stimuli—for each sex class, one stimulus set consisted of adult stimuli, and the other stimulus set consisted of adolescent stimuli. The Kumamoto bonobos only had one previous adolescent female groupmate, one previous adult female groupmate, one previous adolescent male groupmate, and one previous adult male groupmate. Thus, again to maintain even counterbalancing of image presentation and equal degrees of novelty across stimuli, we filled the remaining previous groupmate trials with images of unfamiliar individuals to create fake trials (excluded from all subsequent analyses).

The Planckendael bonobos were presented with two sets of female stimuli, owing to a lack of previous male groupmates for whom photos were available. One stimulus set consisted of adult female stimuli, and the other stimulus set consisted of adolescent stimuli. The Planckendael bonobos saw images of two previous adult female groupmates paired with images of two unknown adult female conspecifics and images of two previous adolescent female groupmates paired with images of two unknown adolescent female conspecifics. We included adolescent bonobo avatars given the limited number of previous adult groupmates for which we could access adequate photos. Adolescent avatars ranged in age from 8.1 to 9.6 y, in line with the period of rapid endocrinological changes in *Pan*, usually beginning between 8 and 10 y old (63, 64). We consider an individual an adult from age 16 y and older, as *Pan* typically reaches adult size and sexual maturity by this age (39, 65–71); but see ref. 63.

For all populations, each image of a previous groupmate was paired with each unfamiliar conspecific image of the same sex and age class to make a stimulus pair. Each stimulus pair was shown twice: once with the previous groupmate on the left and once with the previous groupmate on the right (i.e., previous groupmate A was shown twice with unknown male conspecific A, twice with unknown male conspecific B, and twice with unknown male conspecific C).

Thus, in total, each chimpanzee saw 18 trials with male stimuli and 18 trials with female stimuli (for a total of 36 trials), each Kumamoto bonobo saw 16 trials with male stimuli and 16 trials with female stimuli (for a total of 32 trials), and each Planckendael bonobo saw 16 trials with female stimuli (see *SI Appendix*, Table S13 for a complete list of trials). Within each group, the majority of subjects received an identical stimulus set. However, for the Edinburgh chimpanzees and Planckendael bonobos, some individuals had never lived with a previous groupmate that was included in the original stimulus set given to the other apes in the population; therefore, for these individuals, the original image of the unknown previous groupmate was replaced with an image of a previous groupmate with whom they had lived.

In addition, some of the subjects were unfamiliar with some of the previous groupmates (i.e., they were not yet in the group by the time the previous groupmate died or left the group). Thus, these trials in which both images were unfamiliar to the subject (presented only to maintain even counterbalancing of image presentation) were excluded from analyses.

### Procedure.

At all three facilities, apes voluntarily entered the testing room. At Kumamoto Sanctuary and Planckendael Zoo, apes were temporarily separated from groupmates for testing. At Edinburgh Zoo, apes were not separated, but tests were only administered when other groupmates were at least 1 m away from the subject such that interference was unlikely. Before presenting the test trials, we habituated the Edinburgh and Planckendael apes to the experimental setup by showing each subject at least one set of three images of nonprimate animals with neutral expressions in their natural environments. Kumamoto chimpanzees and bonobos did not require habituation as they had already participated in other eye-tracking studies (39, 65, 66).

The test trials were administered in clusters of three for chimpanzees (twelve clusters total, six with male trials and six with female trials), and in clusters of four for bonobos (four clusters with female trials, and four clusters with male trials). For all populations, in each trial, the images were presented for 3 s following a 0.5-s presentation of a black screen with a fixation cross in the center (intended to center apes’ gaze before trial onset). Within a cluster, trials progressed one immediately following the other for a total duration of 10.5 s per three-trial cluster for chimpanzees and 14 s per four-trial cluster for bonobos. Each cluster featured images of the same sex, and within a cluster, all images of that sex were shown once (each previous groupmate paired once with each unfamiliar individual). The side (left, right) on which the familiar individual was presented was counterbalanced within and across clusters. For Kumamoto bonobos and all chimpanzees (all of which had both male and female stimuli), the presentation alternated between male clusters and female clusters. For bonobos, within a cluster, the presentation alternated between adult and adolescent trials. Cluster order was counterbalanced across subjects. Because participation was voluntary (i.e., apes could walk away from the experimental setup at any time), the number of clusters administered within a day varied between one to six, depending on duration of apes’ attendance and attention at the testing setup. After administering all trials in the predetermined order, we verified that subjects had at least one fixation toward either the familiar or unfamiliar image (see AOI procedure below). After subjects completed their originally assigned trial order, trials that yielded zero fixations to either image were repeated until we had data for a full set of trials for each subject. In total, we tested 848 trials. We excluded 240 “fake” trials that included two completely unknown individuals and 64 “unknown” trials that included previous groupmates whom some of the current participants knew, but who were completely unknown to the subject (i.e., the subject was not yet living in the group before this previous groupmate left the population). Five hundred and forty-four trials remained for analysis, of which 65 featured the subject’s kin (defined as a relatedness coefficient *r* ≥ 0.25, derived from studbook data) and 479 featured nonkin (*r* < 0.25). On average, apes fixated on one or both of the AOIs for 1.06 s (SD = 0.84) of each 3-s trial.

### Administration of Social Relationships Survey and Rater Reliability.

To assess whether previous social relationships have any effects on long-term social memory and attention, we administered a survey of these traits from a minimum of two zoo keepers and/or researchers who had worked with both the study subjects and their previous groupmates (see *SI Appendix* for example). We verified comprehension by each rater using two comprehension questions at the beginning of each survey. Raters were requested not to discuss their responses with other raters, only to assess relationships of dyads they had personally worked with, and to leave any unfamiliar dyads blank. Raters quantified relationships between each subject and each former groupmate as they were the last time the two individuals were in the same group (this was verified with a comprehension question). On a scale of 1 to 5, raters indicated relative dominance between the subject and each former groupmate (with 1 indicating that the subject was very dominant to the groupmate), as well as the dyad’s rates of positive interaction (with 1 representing the highest and 5 the lowest) and negative interaction (again, with 1 representing the highest and 5 the lowest). For each rating, raters indicated their level of confidence (with 1 indicating “complete confidence” and 5 “just guessing”). Overall, confidence assessments were high and we therefore kept all ratings for analyses. Two keepers and/or researchers filled out the survey for the Kumamoto chimpanzees and Planckendael bonobos, four keepers/researchers for the Edinburgh chimpanzees, and one keeper for the Kumamoto bonobos.

We assessed agreement between raters by computing intra-class correlation coefficients [ICC, (67) As a different group of raters rated each population and we planned to assess absolute agreement between raters, we thus calculated the ICC(1,k) for each metric. The results of the ICC analyses and the keeper’s reliability are shown in *SI Appendix*, Table S8. Reliability was high, with all ICC(1,k)’s considered good or excellent: 0.77 for rates of negative social interactions, 0.90 for rates of positive social interactions, and 0.94 for relative dominance (68). As we found strong reliability across indices, we used the mean rating for each metric (for each subject/previous groupmate pair) in the analyses described below.

### Data Scoring and Analysis.

We summed the total fixation duration within each area of interest (AOI) for the entire 3-s trial duration. These AOIs were defined in Tobii Studio as 700 × 700 pixel areas around each of the two images in each trial (i.e., previous groupmate and unfamiliar stranger), and thus each AOI included the image plus a 50-pixel buffer on each side of the image. Fixations were calculated using Tobii Studio’s I-VT Filter, and then the fixation data was exported frame-by-frame from Tobii Studio into TSV files. To measure biases in looking toward the previous groupmate relative to the unfamiliar stranger, we next calculated both raw difference scores (i.e., the sum of fixations to the previous groupmate minus the sum of fixations to an unfamiliar stranger) and a proportional Differential Looking Score (hereafter, DLS; i.e., [Familiar minus Unfamiliar looking time]/[sum of all fixation time]) as dependent variables for each trial. We conducted our analyses in two parts: one using only nonkin trials to exclude kin recognition as an explanation for biased looking toward previous groupmates (see ref. 13, for an equivalent approach), and the second using both kin and nonkin trials. We modeled both measures as dependent variables, as raw difference scores provide a direct measure of the difference in looking time to the familiar individual relative to the unfamiliar individual that also captures variation in overall looking duration. However, to control for variation in overall looking time (40) which may differ across individuals, we also used the DLS, noting that this proportional score, in contrast, amplifies strongly biased looks even on trials when overall looking duration is low. The raw difference scores and DLS variables potentially range from (−3 to 3) and (−1 to 1), respectively.

In Model Set 1, we analyzed whether apes’ attention was biased above-chance toward their previous groupmates, with the prediction that apes would bias their attention toward familiar individuals as an indication of memory of those individuals. Model Set 1 included both kin and nonkin trials, which allowed us to investigate the effect of avatar kinship on apes’ looking biases. In Model Set 2, we tested several hypotheses about the factors that shape memory and attention. Model Set 2a only included nonkin trials, as there were not enough trials with kin to fully explore the effects of these socioecological factors on memory for kin. Initial models comprising the full dataset did not conform to model assumptions; therefore, we reduced our dataset to exclude less informative trials (see below) and repeated analyses of Models 1a and 2a (Models 1b and 2b).

#### General modeling approach.

We fitted linear mixed effects models for both dependent variables (i.e., raw difference score and DLS) using the statistical software R (version 4.0.2; R Core Team 2020). For Model 1a and the Raw Difference Score Model 2b we fitted simple linear mixed models using the *lmer* function from the “lme4” package (69). Here, we used the original intervals for both the Raw Difference Score [−3,3] and DLS [−1,1], so that it was possible to determine whether these scores were significantly different from zero (a model intercept of zero signifies no bias toward the previous groupmates or unfamiliar strangers). For DLS Model 2b, we fitted a generalized linear mixed model using the *glmmTMB* function with a beta distribution from the “glmm” package (70), as this distribution best models proportional scores (71, 72). To do so, the DLS was standardized from its original [−1,1] interval to a (0,1) interval as is required for beta distribution models, which are designed to model proportional scores and require a continuous distribution bounded on this interval.

We used a significance threshold of 0.05 when reporting p-values, and reported trends between 0.05 and 0.1, to avoid arbitrary dichotomization of significance (73). We first used likelihood ratio tests to compare the fit of the full model against the null model, which included only the random effects and control predictors. Then, for Model 1a, the significance of the model intercepts was tested using Satterthwaite’s degrees of freedom method with the “lmerTest” package (74). For Model 2b, we used the *drop1* function provided in the lme4 package to obtain *P*-values for individual terms within these models. We inspected all models to ensure conformity of assumptions of normality and homogeneous distribution of residuals and that models did not have collinear predictors. While we found that no models suffered from issues of collinearity, we discuss below how we overcame other violations of model assumptions.

#### Model Set 1: Discrimination of kin and previous groupmates’ and unfamiliar strangers’ faces.

Model Set 1 tested the prediction of the Long-term Social Memory hypothesis—that apes who remember previous groupmates should show a significant bias in attention toward previous groupmates or unfamiliar conspecifics. Here, we evaluate the model intercept as it represents the population average for our sample, where a significant positive intercept term indicates a bias toward previous groupmates and a significant negative intercept term indicates a bias toward unfamiliar avatars. We included a single categorical test predictor of avatar kinship (i.e., kin or nonkin). We included trial number as a single continuous control predictor to account for any potential habituation effect as trials progress. We z-transformed trial number to center the resulting intercept around the average trial number (16.68, on an original trial interval between 1 and 36). We included subject identity (ID, to account for repeated measures from each subject), and the IDs of the familiar and unfamiliar avatars (as a single dyad measure, to account for potential random variability in preferences for specific individuals) as random intercepts. By releveling avatar kinship (i.e., once with kin as the reference category of the kin vs. nonkin test effect, and then with nonkin as the reference category), we could additionally interpret the intercept term for potential bias in both kin and nonkin trials. The null model was identical to the full model, except that it did not include the test factor avatar kinship or the control predictor z-transformed trial number.

#### Model 2: Predictors of biases in long-term social memory.

In Model Set 2, we tested several hypotheses about the mechanisms that shape long-term social memory and attention. We have named these hypotheses here for ease of referencing. First, to determine whether memory degradation was evident in the time scales captured by our sample (i.e., whether biases became weaker over time; the “Memory Degradation” hypothesis), we included *time apart* (the duration, in years, between testing and the last time the subject was co-housed with the previous groupmate) as a continuous fixed effect. We also included as a continuous fixed effect the subject’s age at separation from the previous groupmate to account for potential age-related effects on recognition. We included the continuous fixed effect of *time together* (the duration, in years, of co-housing between the subject and the previous groupmate) to test the Familiarity prediction of the “Relationship Memory hypothesis” that biases will be greater for dyads that had spent more time together. Finally, we included several social factors to test the predictions of the Relationship Memory hypothesis that apes’ memory incorporates relationship content: an interaction between species and avatar sex [in line with previous findings that apes bias attention toward familiar members of the dominant sex, e.g., male chimpanzees or female bonobos: (38), index of positive social interaction, index of negative social interaction, and index of relative dominance]. As in Model Set 1, we included z-transformed trial number as a control predictor to account for potential habituation over trials, as well as subject sex as a control predictor. We further included the age class of the previous groupmate at the time of separation as a categorical control predictor to account for potential biases in attention toward individuals of a certain age group. Here, we used age class as a categorical rather than continuous variable because for some previous groupmates, we did not have exact dates of birth and thus could not accurately calculate the exact age at separation. These age categories were based on biologically based categorizations from the literature on wild chimpanzees and bonobos [adolescent = 8–15 y, adult = 16–35 y, older adult = 35+ y; (75–77)]. Finally, we included population, dummy-coded as European apes (Edinburgh chimpanzee and Planckendael bonobos) and Kumamoto apes (Kumamoto Sanctuary chimpanzees and bonobos), as a categorical test predictor because previous eye-tracking research with these populations identified differences between conspecific populations (38). As random intercepts, we included subject identity (ID, to account for repeated measures from each subject), and the IDs of the familiar and unfamiliar avatars (i.e., ID of each dyad pair, to account for potential random variability in preferences for specific individuals). Null models included all random intercepts and control predictors (trial number, population, subject sex, subject age, avatar age class, and time together) while lacking all aforementioned test predictors (index of positive social interaction, index of negative social interaction, index of relative dominance, the interaction between species and avatar sex, and time apart).

#### Models 1 and 2 with restricted datasets: Predictors of biases in true recognition of previous groupmates.

We found the distribution of model residuals in Model 2a to violate assumptions of normality. Upon visual inspection, we found the source of residual skews to derive from a large number of trials (196 of 463 nonkin trials) in which apes only fixated on a single avatar rather than both (i.e., DLS = 1 [only fixations toward the previous groupmate] or −1 [only fixations toward the unknown conspecific]). We subsequently removed these trials given that trials in which subjects have not observed both avatars may not be informative for discrimination (see refs. 42 and 43 for similar exclusion criteria). The remaining 267 nonkin trials in which subjects looked at both avatars constituted our refined dataset, with which we fitted Model 2b. We also refitted Model 1a with a reduced dataset that excluded trials in which apes only fixated on a single avatar rather than both (210 out of 524 trials with kin and nonkin; 314 trials remaining, included in Model 1b). As the majority of removed trials (111 of 210 kin and nonkin trials) consisted of fixation only upon the familiar groupmate, we note that their removal makes Models 1b and 2b even more conservative against our predictions.

## Supplementary Material

Appendix 01 (PDF)

Dataset S01 (CSV)

Dataset S02 (CSV)

Dataset S03 (XLSX)

## Data Availability

All study data are available in Github (https://github.com/LauraLewis15/Ape-Long-Term-Social-Memory-Data) (41) and supporting information.
