# Supplementary material for: Bonobos and chimpanzees remember familiar conspecifics for decades
Source: Proc Natl Acad Sci U S A. 2023 Dec 18;120(52):e2304903120. doi: 10.1073/pnas.2304903120 (PMC10756267; doi:10.1073/pnas.2304903120)
Supplement: Supplementary file 1 — Appendix 01 (PDF) [file pnas.2304903120.sapp.pdf]

Supporting Information for

Bonobos and chimpanzees remember familiar conspecifics for decades

Laura S. Lewis[1,2,*], Erin G. Wessling[1,2], Fumihiro Kano[3,4], Jeroen M. G. Stevens[5,6], Josep Call[2], & Christopher Krupenye[2,7*]

1. Department of Human Evolutionary Biology, Harvard University, Cambridge, MA, 02138, USA
2. School of Psychology & Neuroscience, University of St Andrews, St Andrews, KY16 9AX, UK
3. Kumamoto Sanctuary, Wildlife Research Center, Kyoto University, Kumamoto, 862-0911, Japan
4. Center for the Advanced Study of Collective Behavior, University of Konstanz, Konstanz, 78457, Germany
5. Behavioural Ecology and Ecophysiology, Department of Biology, University of Antwerp, Antwerp, BE-2000, Belgium
6. Centre for Research and Conservation, Royal Zoological Society of Antwerp, Antwerp, 2018, Belgium
7. Department of Psychological & Brain Sciences, Johns Hopkins University, Baltimore, MD, 21218, USA

*Correspondence: laura.lewis889@gmail.com (LSL) or krupenye@jhu.edu (CK)

This PDF file includes:

Supporting text
Tables S1 to S21
Keeper Survey Example

Other supporting materials for this manuscript include the following:

Datasets S1 to S3

Results:

Analyses with DLS

The full-null model comparison for **Model Set 1a** was not significant (**DLS:** χ^2 = 0.0005, p = 0.983), indicating that our single test predictor - whether the former groupmate was the subject's kin - did not influence looking biases. **Model Set 1a** revealed a positive and significant intercept term with non-kin as the reference category of the kin vs. non-kin test effect (**DLS:** estimate = 0.112 ± 0.039 (SE), p = 0.009), indicating a significant looking bias toward non-kin former groupmates. Within the same **Model Set 1a**, when kin was set as the reference category, the intercept term was not significant for the DLS model (**DLS:** estimate = 0.109 ± 0.099 (SE), p = 0.268).

Re-fitting **Model Set 1b** to the refined dataset produced identical results, as we found no significant difference in looking patterns between trials with former groupmates that were kin versus non-kin (**DLS:** estimate = - 0.031 ± 0.085 (SE), p = 0.714). The intercept term also remained significant for non-kin trials (**DLS:** estimate = 0.136 ± 0.044 (SE), p = 0.005, see **Figure 2**), with apes fixating on average for 0.25 seconds (~14%) longer toward images of former groupmates than toward strangers. For kin trials, we did not find a significant intercept term (**DLS:** estimate = 0.105 ± 0.084 (SE), p = 0.214).

Predictors of attentional biases

Full-null model comparisons for **Model Set 2b** were not significant (**DLS:** χ^2 = 5.212, p = 0.517). There was no significant interaction between species and avatar sex, and therefore we dropped this interaction term and re-fitted the models. Critically, the effect of time apart was not significant, suggesting that memory did not significantly differ across the durations captured within the non-kin dataset (maximum = 9.54 years). The effect of population on looking bias was not significant for **DLS Model Set 2b** (**DLS:** estimate = 0.330 ± 0.199 (SE), p = 0.097). Finally, we found an effect of trial number (**DLS:** estimate = - 0.180 ± 0.065 (SE), p = 0.005), indicating a decline in overall attention toward avatars as trials progressed (see **Tables A7, A8)**.

Table S1: Characteristics of study participants

Individual	Species	Sex	Date of Birth	Age	Facility
Frek	Chimpanzee	M	10/21/93	24.73	Edinburgh
Liberius	Chimpanzee	M	1/20/99	19.48	Edinburgh
Louis	Chimpanzee	M	7/26/76	41.97	Edinburgh
Qafzeh	Chimpanzee	M	3/31/92	26.29	Edinburgh
Rene	Chimpanzee	M	2/21/93	25.39	Edinburgh
Velu	Chimpanzee	M	6/24/14	4.06	Edinburgh
Edith	Chimpanzee	F	4/11/96	22.26	Edinburgh
Eva	Chimpanzee	F	12/9/80	37.60	Edinburgh
Kilimi	Chimpanzee	F	2/20/93	25.40	Edinburgh
Iroha	Chimpanzee	F	12/28/03	15.05	Kumamoto Sanctuary
Mizuki	Chimpanzee	F	1/14/95	24.00	Kumamoto Sanctuary
Hatsuka	Chimpanzee	F	4/20/89	29.74	Kumamoto Sanctuary
Misaki	Chimpanzee	F	2/3/82	36.95	Kumamoto Sanctuary
Natsuki	Chimpanzee	F	11/27/91	27.13	Kumamoto Sanctuary
Zamba	Chimpanzee	M	10/28/72	46.21	Kumamoto Sanctuary
Habari	Bonobo	M	1/29/06	12.64	Planckendael
Kikongo	Bonobo	M	1/29/14	4.64	Planckendael
Lina	Bonobo	F	7/28/85	33.15	Planckendael
Djanoa	Bonobo	F	3/27/95	23.49	Planckendael
Nayoki	Bonobo	F	3/24/12	6.49	Planckendael

Vijay	Bonobo	M	12/28/03	15.13	Kumamoto Sanctuary
Junior	Bonobo	M	1/14/95	24.09	Kumamoto Sanctuary
Lolita	Bonobo	F	4/20/89	29.82	Kumamoto Sanctuary
Connie Lenore	Bonobo	F	2/3/82	37.03	Kumamoto Sanctuary
Ikela	Bonobo	F	11/27/91	27.22	Kumamoto Sanctuary
Louise	Bonobo	F	10/28/72	46.30	Kumamoto Sanctuary

Table S2: Characteristics of participant groups at time of testing

**Facility**	**Total # of Individuals**	**# Males**	**# Females**
Edinburgh Zoo (Chimpanzees)	15	8	7
Planckendael Zoo (Bonobos)	13	7	6
Kumamoto Sanctuary (Bonobos)	6	2	4
Kumamoto Sanctuary (Chimpanzees)	6	1	5

Table S3: Duration Between Testing and Last Time of Co-Housing
Duration, in years, between testing and the last time the participants were co-housed with their previous groupmates.

Population	Previous Groupmate	Sex	Age Class	Years Apart (from each subject)
Edinburgh	Emma	F	Adult	3.15 (all subjects)
Edinburgh	Lyndsey	F	Adult	5.91 (all subjects)
Edinburgh	Pearl	F	Adult	2.3 (all subjects)
Edinburgh	Bram	M	Adult	8.15 (all subjects)
Edinburgh	Claus	M	Adult	6.96 (all subjects)
Edinburgh	Kindia	M	Adult	0.73 (all subjects)
Edinburgh	Ricky	M	Adult	6.99 (all subjects)
Planckendael Bonobos	Hermien	F	Adult	9.54 (all subjects)
Planckendael Bonobos	Kumbuka	F	Adult	3.57 (Kikongo)
Planckendael Bonobos	Jasiri	F	Adolescent	9.15 (all subjects)
Planckendael Bonobos	Jill	F	Adult	3.57 (Kikongo)
Planckendael Bonobos	Lingoye	F	Adolescent	3.39 (all subjects)
Planckendael Bonobos	Lomela	F	Adult	10.4 (all subjects)
Planckendael Bonobos	Zuani	F	Adult	3.57 (Kikongo)
Kumamoto Chimpanzees	Loi	M	Adult	4.49 (all subjects)
Kumamoto Chimpanzees	Tsubaki	M	Adult	4.49 (all subjects)
Kumamoto Bonobos	Kalli	F	Adolescent	5.48 (all subjects)
Kumamoto Bonobos	Loretta	F	Adult	26.20 (Louise)<br>5.48 (all other subjects)
Kumamoto Bonobos	Erin	M	Adult	26.20 (Louise)<br>5.48 (all other subjects)

Kumamoto Bonobos	Makasi	M	Adolescent	5.48 (all subjects)

Table S4: Raw Difference Scores Model 1a (Full Dataset)
Predictors of biases in attention toward faces of previous groupmates, with non-kin as a reference category. The intercept effect refers to data from non-kin trials. Raw difference was used as the dependent measure. Subject and avatar dyad were included as random intercepts. P values less than 0.05 are bolded. Estimates and SEs are taken from the model summary.

**Fixed Effects**	**Estimate**	**Std. Error**	**df**	**P value**
Intercept	0.244	0.072	23.218	**0.002**
Z-Transformed Trial Number	-0.036	0.039	481.934	0.365
Kin_Non-Kin	0.001	0.136	289.258	0.992

Table S5: DLS Model 1a (Full Dataset)
Predictors of biases in attention toward faces of previous groupmates, with non-kin as a reference category. The intercept effect refers to data from non-kin trials. DLS was used as the dependent measure. Subject and avatar dyad were included as random intercepts. P values less than 0.05 are bolded. Estimates and SEs are taken from the model summary.

**Fixed Effects**	**Estimate**	**Std. Error**	**df**	**P value**
Intercept	0.112	0.039	24.768	**0.009**
Z-Transformed Trial Number	0.012	0.032	514.671	0.716
Kin_Non-Kin	-0.002	0.104	254.531	0.983

Table S6: Raw Difference Scores Model 1b (Reduced Dataset with looks to both AOIs)
Predictors of biases in attention toward faces of previous groupmates, with non-kin as a reference category. The intercept effect refers to data from non-kin trials. Raw difference was used as the dependent measure. Subject and avatar dyad were included as random intercepts. P values less than 0.05 are bolded. Estimates and SEs are taken from the model summary.

**Fixed Effects**	**Estimate**	**Std. Error**	**df**	**P value**
Intercept	0.248	0.084	26.44	**0.006**
Z-Transformed Trial Number	-0.135	0.048	310.10	**0.005**
Kin_Non-Kin	0.0003	0.153	258.90	0.998

Table S7: DLS Model 1b (Reduced Dataset with looks to both AOIs)
Predictors of biases in attention toward faces of previous groupmates, with non-kin as a reference category. The intercept effect refers to data from non-kin trials. DLS was used as the dependent measure. Subject and avatar dyad were included as random intercepts. P values less than 0.05 are bolded. Estimates and SEs are taken from the model summary.

**Fixed Effects**	**Estimate**	**Std. Error**	**df**	**P value**
Intercept	0.136	0.044	25.69	**0.005**
Z-Transformed Trial Number	-0.065	0.027	311.53	**0.016**
Kin_Non-Kin	-0.031	0.085	235.49	0.714

Table S8: Results of ICC Analyses for Keeper Ratings
The three traits included in the Keeper/Researcher Survey showing the reliability of ratings, using intra-class correlation coefficients (ICCs). The ICC(1,k) reports the absolute agreement between the raters (different raters for each population).

**Trait**	**Measure**	**ICC(1,k)**
Rates of Positive Social Interactions	Rates of grooming, playing, and proximity	0.90
Rates of Negative Social Interactions	Rates of aggression targeting one another	0.77
Relative Dominance	Relative dominance status between participant and previous groupmates	0.94

Table S9: Raw Difference Scores Model 2b (Reduced Dataset with non-kin trials and looks to both AOIs)
Predictors of biases in attention toward faces of previous groupmates. Raw difference was used as the dependent measure. Subject and avatar dyad were included as random intercepts. P values less than 0.05 are bolded. Estimates and SEs are taken from the model summary; degrees of freedom and P values are taken from the
drop1 Chi-sq output.

**Fixed Effects**	**Estimate**	**Std. Error**	**df**	**P value**
Intercept	0.157	0.514	1	0.999
Rates of Positive Interactions	-0.159	0.081	1	**0.049**
Rates of Negative Interactions	0.031	0.065	1	0.642
Relative Dominance	0.021	0.058	1	0.718
Avatar Sex (M)	-0.007	0.138	1	0.959

Species (Chimp)	0.076	0.210	1	0.718
Subject Sex (M)	0.052	0.113	1	0.647
Population (Dummy)	0.470	0.167	1	**0.006**
Years Apart	0.018	0.025	1	0.492
Years Together	-0.005	0.010	1	0.608
Trial Number (Z-Transformed)	-0.163	0.054	1	**0.003**
Subject Age	0.004	0.007	1	0.485
Avatar Age(Old Adult)	-0.080	0.225	2	0.938

Table S10: DLS Model 2b (Reduced Dataset with non-kin trials and looks to both AOIs)
Predictors of biases in attention toward faces of previous groupmates. DLS was used as the dependent measure. Subject and avatar dyad were included as random intercepts. P values less than 0.05 are bolded, p values between 0.05 and 0.1 are italicized. Estimates and SEs are taken from the model summary; degrees of freedom and P
values are taken from the drop1 Chi-sq output.

**Fixed Effects**	**Estimate**	**Std. Error**	**df**	**P value**
Intercept	0.025	0.615	1	0.968
Rates of Positive Interactions	-0.134	0.096	1	0.163
Rates of Negative Interactions	0.054	0.079	1	0.495
Relative Dominance	0.056	0.068	1	0.418
Avatar Sex (M)	0.121	0.160	1	0.453
Species (Chimp)	0.231	0.246	1	0.350

Subject Sex (M)	0.014	0.135	1	0.919
Population (Dummy)	0.330	0.198	1	*0.097*
Years Apart	0.016	0.029	1	0.597
Years Together	-0.006	0.012	1	0.632
Trial Number (Z-Transformed)	-0.180	0.064	1	**0.005**
Subject Age	0.006	0.008	1	0.497
Avatar Age (Old Adult)	-0.316	0.263	2	0.348

Table S11: Total Attention Toward Both Stimuli
Predictors of total attention toward both stimuli (i.e., attention to former groupmate + attention to stranger) presented on the screen (seconds), including data from non-kin trials with at least one fixation toward each AOI. Subject and avatar dyad were included as random intercepts. P values less than 0.05 are bolded. Estimates and SEs are taken from the model summary.

**Fixed Effects**	**Estimate**	**Std. Error**	**df**	**P value**
Intercept	1.010	0.089	17.362	**<0.001**
Population	0.900	0.153	19.293	**<0.001**
Z-Transformed Trial Number	-0.104	0.042	218.265	**0.0131**

Table S12: Total Number of Lifetime Groupmates

Total number of individuals that each participant has lived with across their lifetime. This value is the sum of the number of groupmates at the time of testing plus all past groupmates.

Participant	Species	Facility	Lifetime # of Groupmates	# of Groupmates at Time of Testing
Frek	Chimpanzee	Edinburgh	23	15
Liberius	Chimpanzee	Edinburgh	24	15
Louis	Chimpanzee	Edinburgh	41	15
Qafzeh	Chimpanzee	Edinburgh	30	15
Rene	Chimpanzee	Edinburgh	23	15
Velu	Chimpanzee	Edinburgh	19	15
Edith	Chimpanzee	Edinburgh	23	15
Eva	Chimpanzee	Edinburgh	23	15
Kilimi	Chimpanzee	Edinburgh	28	15
Iroha	Chimpanzee	Kumamoto Sanctuary	7	5
Mizuki	Chimpanzee	Kumamoto Sanctuary	7	5
Hatsuka	Chimpanzee	Kumamoto Sanctuary	7	5
Misaki	Chimpanzee	Kumamoto Sanctuary	7	5
Natsuki	Chimpanzee	Kumamoto Sanctuary	7	5
Zamba	Chimpanzee	Kumamoto Sanctuary	7	5
Habari	Bonobo	Planckendael	20	14

Kikongo	Bonobo	Planckendael	22	14
Lina	Bonobo	Planckendael	51	14
Djanoa	Bonobo	Planckendael	29	14
Nayoki	Bonobo	Planckendael	14	14
Vijay	Bonobo	Kumamoto Sanctuary	17	5
Junior	Bonobo	Kumamoto Sanctuary	21	5
Lolita	Bonobo	Kumamoto Sanctuary	29	5
Connie Lenore	Bonobo	Kumamoto Sanctuary	31	5
Ikela	Bonobo	Kumamoto Sanctuary	30	5
Louise	Bonobo	Kumamoto Sanctuary	36	5

Table S13: Numbers of Unique Stimuli for Each Ape Population
Exact numbers of unique stimuli shown to each ape population included in the study, broken down by sex of avatars included in stimuli images. 'G' refers to adult previous groupmates and 'S' refers to adult strangers. 'AG' refers to adolescent previous groupmates and 'AS' refers to adolescent strangers. All pairs were presented in two orientations (once with the groupmate on the left, and once with the groupmate on the right), resulting in trial numbers twice that of stimulus pair numbers. Valid trials refer to those in which a previous groupmate and stranger were presented together. These constituted the dataset included in our analyses. Where the target number of groupmate stimuli was not available, some stranger-stranger pairs were used to ensure equal presentations of all test stimuli within the pair.

Population	Number of Male Pairs	Number of Female Pairs	Number of Valid Trials	Total Number of Trials
Edinburgh Chimpanzees	3G x 3S = 9 valid pairs	3G x 3S = 9 valid pairs	36 (18 trials with male stimuli, 18 trials with female stimuli)	36 (18 trials with male stimuli, 18 trials with female stimuli)
Planckendael Bonobos	0	2G x 2G = 4 valid pairs<br>2AG x 2AS = 4 valid pairs	16 (8 trials with adult female stimuli, 8 trials with adolescent female stimuli)	16 (8 trials with adult female stimuli, 8 trials with adolescent female stimuli)
Kumamoto Chimpanzees	(1G + 2S) x 3S = 3 valid pairs and 6 filler pairs	(1G + 2S) x 3S = 3 valid pairs and 6 filler pairs	12 (6 trials with male stimuli, and 6 trials with female stimuli)	36 (18 trials with male stimuli, 18 trials with female stimuli)
Kumamoto Bonobos	(1G + 1S) x 2S = 2 valid pairs and 2 filler pairs<br>(1AG + 1AS) x 2S = 2 valid pairs and 2 filler pairs	(1G + 1S) x 2S = 2 valid pairs and 2 filler pairs<br>(1AG + 1AS) x 2S = 2 valid pairs and 2 filler pairs	16 (8 trials with male stimuli, and 8 trials with female stimuli)	32 (16 trials with male stimuli, 16 trials with female stimuli)

Table S14: Raw Difference Scores Model 1 excluding Kumamoto Sanctuary Data (European Dataset with looks to both AOIs)

Predictors of biases in attention toward faces of previous groupmates, with non-kin as a reference category. The intercept effect refers to data from non-kin trials. Raw difference was used as the dependent measure. Subject and avatar dyad were included as random intercepts. P values less than 0.05 are bolded. Estimates and SEs are taken from the model summary.

Fixed Effects	Estimate	Std. Error	df	P value
Intercept	0.179	0.589	11.297	0.766
Z-Transformed Trial Number	-0.157	0.051	167.144	**0.002**
Kin_Non-Kin	0.144	0.213	172.127	0.500

Table S15: DLS Model 1 excluding Kumamoto Sanctuary Data (European Dataset with looks to both AOIs)

Predictors of biases in attention toward faces of previous groupmates, with non-kin as a reference category. The intercept effect refers to data from non-kin trials. DLS was used as the dependent measure. Subject and avatar dyad were included as random intercepts. P values less than 0.05 are bolded. Estimates and SEs are taken from the model summary.

Fixed Effects	Estimate	Std. Error	df	P value
Intercept	0.030	0.053	11.759	0.581
Z-Transformed Trial Number	-0.088	0.037	176.042	**0.017**
Kin_Non-Kin	0.009	0.154	181.360	0.951

Table S16: Raw Difference Scores Model 1 using only Kumamoto Sanctuary Data (Kumamoto Dataset with looks to both AOIs)

Predictors of biases in attention toward faces of previous groupmates, with non-kin as a reference category. The intercept effect refers to data from non-kin trials. Raw difference was used as the dependent measure. Subject and avatar dyad were included as random intercepts. P values less than 0.05 are bolded. Estimates and SEs are taken from the model summary.

**Fixed Effects**	**Estimate**	**Std. Error**	**df**	**P value**
Intercept	0.565	0.132	14.319	**0.0007**
Z-Transformed Trial Number	-0.135	0.088	123.452	0.128
Kin_Non-Kin	0.174	0.218	77.297	0.428

Table S17: DLS Model 1 using only Kumamoto Sanctuary Data (Kumamoto Dataset with looks to both AOIs)

Predictors of biases in attention toward faces of previous groupmates, with non-kin as a reference category. The intercept effect refers to data from non-kin trials. DLS was used as the dependent measure. Subject and avatar dyad were included as random intercepts. P values less than 0.05 are bolded. Estimates and SEs are taken from the model summary.

**Fixed Effects**	**Estimate**	**Std. Error**	**df**	**P value**
Intercept	0.179	0.080	24.265	**0.035**
Z-Transformed Trial Number	-0.049	0.039	123.770	0.207
Kin_Non-Kin	0.097	0.091	53.037	0.289

Analyses with Trials with Total Looking Time Over 400 ms

In humans, recognition of familiar faces occurs as fast as 380 ms (1). We thus tested whether the results from **Model Sets 1** and **2** were consistent when only including trials with > 400 ms total looking time toward the screen (both inside and outside of the AOIs). We found that results were consistent across both Model Sets, and that significant patterns were even more pronounced within these trials. Below are the results from these analyses.

Table S18: Raw Difference Scores Model 1 (Full Screen Total Looking Time Dataset)
Predictors of biases in attention toward faces of previous groupmates, with non-kin as a reference category. The intercept effect refers to data from non-kin trials. Raw difference score was used as the dependent measure. Subject and avatar dyad were included as random intercepts. P values less than 0.05 are bolded. Estimates and SEs are taken from the model summary.

Fixed Effects	Estimate	Std. Error	df	P value
Intercept	0.250	0.082	21.458	**0.006**
Z-Transformed Trial Number	-0.143	0.056	481.934	0.011
Kin_Non-Kin	-0.273	0.261	289.258	0.298

Table S19: DLS Model 1 (Full Screen Total Looking Time Dataset)
Predictors of biases in attention toward faces of previous groupmates, with non-kin as a reference category. The intercept effect refers to data from non-kin trials. DLS was used as the dependent measure. Subject and avatar dyad were included as random intercepts. P values less than 0.05 are bolded. Estimates and SEs are taken from the model summary.

Fixed Effects	Estimate	Std. Error	df	P value
Intercept	0.134	0.044	21.468	**0.007**
Z-Transformed Trial Number	-0.069	0.031	257.983	**0.027**
Kin_Non-Kin	-0.183	0.145	240.04	0.207

Table S20: Raw Difference Scores Model 2 (Full Screen Total Looking Time Dataset)
Predictors of biases in attention toward faces of previous groupmates. Raw difference was used as the dependent measure. Subject and avatar dyad were included as random intercepts. P values less than 0.05 are bolded. Estimates and SEs are taken from the model summary; degrees of freedom and P values are taken from the drop1 Chi-sq output.

**Fixed Effects**	**Estimate**	**Std. Error**	**df**	**P value**
Intercept	0.099	0.595	1	0.999
Rates of Positive Interactions	-0.239	0.089	1	**0.008**
Rates of Negative Interactions	0.059	0.075	1	0.424
Relative Dominance	0.042	0.065	1	0.518
Avatar Sex (M)	-0.101	0.158	1	0.526
Species (Chimp)	0.205	0.237	1	0.391
Subject Sex (M)	0.025	0.126	1	0.842
Population (Dummy)	0.425	0.188	1	**0.026**
Years Apart	0.028	0.029	1	0.359
Years Together	-0.003	0.013	1	0.813
Trial Number (Z-Transformed)	-0.207	0.061	1	**0.0008**
Subject Age	0.006	0.008	1	0.388
Avatar Age(Old Adult)	-0.119	0.262	2	0.889

Table S21: DLS Model 2 (Full Screen Total Looking Time Dataset)
Predictors of biases in attention toward faces of previous groupmates. DLS was used as the dependent measure. Subject and avatar dyad were included as random intercepts. P values less than 0.05 are bolded, p values between 0.05 and 0.1 are italicized. Estimates and SEs are taken from the model summary; degrees of freedom and P values are taken from the drop1 Chi-sq output.

**Fixed Effects**	**Estimate**	**Std. Error**	**df**	**P value**
Intercept	-0.229	0.615	1	
Rates of Positive Interactions	-0.188	0.096	1	*0.073*
Rates of Negative Interactions	0.094	0.079	1	0.273
Relative Dominance	0.083	0.068	1	0.264
Avatar Sex (M)	0.027	0.160	1	0.877
Species (Chimp)	0.328	0.246	1	0.208
Subject Sex (M)	-0.011	0.135	1	0.939
Population (Dummy)	0.256	0.198	1	0.225
Years Apart	0.024	0.029	1	0.469
Years Together	0.004	0.012	1	0.785
Trial Number (Z-Transformed)	-0.211	0.064	1	**0.003**
Subject Age	0.009	0.008	1	0.303
Avatar Age (Old Adult)	-0.309	0.263	2	0.403

Supporting Information References

1. M. Ramon, S. Caharel, B. Rossion. The speed of recognition of personally familiar faces. *Perception* **40**, 437–449 (2011).

Great Ape Survey

Thank you very much for taking the time to fill out this survey! Your answers on this survey will be extremely helpful for our eye-tracking research. Completion of this survey implies consent to participate in this research. Please print this survey to fill it out, and once you have filled it out you are welcome to either scan it or take a picture to email it back to Laura Lewis (laura_lewis@g.harvard.edu). Thank you so much!

Instructions:

a. Please fill out the survey on your own, without input from others
b. Please read each pairing carefully before responding
c. If you do not remember the answer to a specific question, please write "I don't know"
d. Please let us know if there is anything important we should know about any specific relationship or previous groupmate in the notes section provided after each table
e. Please circle whole numbers on each scale; please do not circle in between numbers.
f. If you did not work with an animal, please check the box "I never worked with this animal" when filling out the absolute dominance table. You are then welcome to leave the rows with this individual blank in each table.
g. Please identify the dominance status and relationship of each ape as they were **the last time the previous groupmates and study participants were in the same group (i.e., shortly before they died or were transferred to another facility).** We know that these individuals were not all in the group at the same time.
h. For each question, please indicate your level of confidence in your answer to the question on a scale from 1 – 5 (1 = completely confident; 3 = somewhat confident; 5 = just guessing)

Questions:

1) When did you begin working with the bonobos at Planckendael Zoo? (month and year, if you remember):

____

2) What are the total number of months that you have worked with this great ape population?

____

3) Are you a full-time keeper working with apes? If not, what other species do you currently work with?

____

4) **Comprehension questions (these questions are added just to ensure that the instructions are clear):**

a. Based on the instructions above, for what period should you attribute absolute dominance ranking for previous group members on this survey? Circle one:

i. Now
ii. In general
iii. The last time the previous groupmates and study participants were in the same group

b. Based on question 5 below, what number should you attribute to a bonobo who is highest ranking on this survey? Circle one:

- **i.** 1
- **ii.** 3
- **iii.** 5

5) Please identify the absolute dominance status of each individual for the last time that the previous groupmates and study participants were in the same group, by circling a whole number on the scale from 1 -5 (1 = most dominant in the group; 3 = mid-ranking; 5 = most subordinate in the group). Absolute dominance status refers to their position in the dominance hierarchy with respect to their entire group. It is ok to choose the same absolute dominance status for more than one group member.

Absolute Dominance Status: 1 = most dominant, 5 = most subordinate
Confidence Level: 1 = completely confident; 3 = somewhat confident; 5 = just guessing

	Absolute Dominance Status:	I never worked with this animal:	Confidence level for answer:
**Hermien:** Date of departure: February, 2010	1 2 3 4 5	☐	1 2 3 4 5
**Lingoye:** Date of departure: September, 2016	1 2 3 4 5	☐	1 2 3 4 5
**Lomela:** Date of departure: September, 2009	1 2 3 4 5	☐	1 2 3 4 5
**Jasiri:** Date of departure: December, 2010	1 2 3 4 5	☐	1 2 3 4 5

Please use the space below to write any notes about the absolute dominance status of these previous groupmates. Please let us know if there is anything important we should know about any of these individuals. You are also welcome to write a few notes on why you chose the specific absolute dominance status for any previous groupmate.

6) Please fill out the table below using the scales described below. Circle one whole number on each scale. Please fill out these ratings of relative dominance and social relationship as they were **the last time the two individuals were in the same group (usually the time the one called 'groupmate' died or was transferred to another facility).**

a. **Relative Dominance Status (scale from 1-5):**

   i. 1 = Participant is very dominant to groupmate
   ii. 2 = Participant is dominant to groupmate
   iii. 3 = Participant has roughly equal dominance status with groupmate
   iv. 4 = Participant is subordinate to groupmate
   v. 5 = Participant is very subordinate to groupmate

b. **Positive Social Interactions (scale from 1-5):**

   i. 1 = High rates of positive interactions (lots of grooming and playing, often in close proximity)
   ii. 3 = Intermediate rates of positive interactions (some grooming and playing, sometimes in close proximity)
   iii. 5 = Low rates of positive interactions (very little grooming or playing, rarely in close proximity)

c. **Negative Social Interactions (scale from 1-5):**

   i. 1 = High rates of negative interactions (lots of aggression targeting one another)
   ii. 3 = Intermediate rates of negative interactions (limited aggression targeting one another)
   iii. 5 = Low rates of negative interactions (rare aggression targeting one another)

d. **Confidence Level (scale from 1-5):**

   i. 1 = Fully confident about accuracy of answer
   ii. 3 = Somewhat confident, but not fully
   iii. 5 = Not confident at all about accuracy of answer

Partic-ipant	Group-mate	**Relative Dominance Status** (1 = participant very dominant to groupmate, 5 = participant very subordinate to groupmate)	**Confidence Level** (1 = completely confident in answer for relative dominance, 5 = just guessing)	**Positive Social Interactions** (1 = High rates of positive interactions, 5 = Low rates of positive interactions)	**Negative Social Interactions** (1 = High rates of negative interactions, 5 = Low rates of negative interactions)	**Confidence Level** (1 = completely confident in answer for social interactions, 5 = just guessing)
**Djanoa**	**Hermien**	1 2 3 4 5	1 2 3 4 5	1 2 3 4 5	1 2 3 4 5	1 2 3 4 5
**Djanoa**	**Lingoye**	1 2 3 4 5	1 2 3 4 5	1 2 3 4 5	1 2 3 4 5	1 2 3 4 5
**Djanoa**	**Lomela**	1 2 3 4 5	1 2 3 4 5	1 2 3 4 5	1 2 3 4 5	1 2 3 4 5
**Djanoa**	**Jasiri**	1 2 3 4 5	1 2 3 4 5	1 2 3 4 5	1 2 3 4 5	1 2 3 4 5
**Lina**	**Hermien**	1 2 3 4 5	1 2 3 4 5	1 2 3 4 5	1 2 3 4 5	1 2 3 4 5
**Lina**	**Lingoye**	1 2 3 4 5	1 2 3 4 5	1 2 3 4 5	1 2 3 4 5	1 2 3 4 5
**Lina**	**Lomela**	1 2 3 4 5	1 2 3 4 5	1 2 3 4 5	1 2 3 4 5	1 2 3 4 5
**Lina**	**Jasiri**	1 2 3 4 5	1 2 3 4 5	1 2 3 4 5	1 2 3 4 5	1 2 3 4 5
**Habari**	**Hermien**	1 2 3 4 5	1 2 3 4 5	1 2 3 4 5	1 2 3 4 5	1 2 3 4 5
**Habari**	**Lingoye**	1 2 3 4 5	1 2 3 4 5	1 2 3 4 5	1 2 3 4 5	1 2 3 4 5
**Habari**	**Lomela**	1 2 3 4 5	1 2 3 4 5	1 2 3 4 5	1 2 3 4 5	1 2 3 4 5
**Habari**	**Jasiri**	1 2 3 4 5	1 2 3 4 5	1 2 3 4 5	1 2 3 4 5	1 2 3 4 5
**Nayoki**	**Lingoye**	1 2 3 4 5	1 2 3 4 5	1 2 3 4 5	1 2 3 4 5	1 2 3 4 5

Please use the space below to write any notes about the relative dominance statuses or social relationships that you rated in the table above. Please let us know if there is anything important we should know about any previous relationships.

You have completed this survey! Thank you very much for taking the time and energy to fill out this survey, we greatly appreciate your input. Please take a picture or scan this completed survey and email it back to Laura Lewis at laura_lewis@g.harvard.edu

There is a chance that it would be helpful for us to contact you if we have questions regarding the results of this survey. If you are willing to be contacted in the future regarding this survey, please sign below:

Email: ____

Signature: ____
